# Can filled pauses be represented as linguistic items? Investigating the effect of exposure on the perception and production of *um*

**DOI:** 10.1177/00238309211011201

**Published:** 2021-05-24

**Authors:** Minna Kirjavainen, Ludivine Crible, Kate Beeching

**Affiliations:** English Language and Linguistics, University of the West of England, UK; F.R.S.-FNRS & Université catholique de Louvain, Belgium; English Language and Linguistics, University of the West of England, UK

**Keywords:** Filled pauses, *um*, input, sentence judgment, sentence repetition, English

## Abstract

The current paper presents three studies that investigated the effect of exposure on the mental representations of filled pauses (*um/uh*). In Study 1, a corpus analysis identified the frequency of co-occurrence of filled pauses with words located immediately before or after them in naturalistic spoken adult British English (BNC2014). Based on the collocations identified in Study 1, in Study 2, 22 native British English-speaking adults heard sentences in which the location of filled pauses and the co-occurring words were manipulated and the participants were asked to judge the acceptability of the sentences heard. Study 3 was a sentence recall experiment in which we asked 29 native British English adults to repeat a similar set of sentences as used in Study 2. We found that frequency-based distributional patterns of filled pauses (Study 1) affected the sentence judgments (Study 2) and repetition accuracy (Study 3), in particular when the filled pause followed its collocate. Thus, the current study provides converging evidence for the account maintaining that filled pauses are linguistic items. In addition, we suggest filled pauses in certain locations could be considered as grammatical items, such as suffixes.

## 1 Introduction

Spontaneous speech production is characterized as a demanding cognitive task facing various sources of pressure often with little preparation as to what is being said or how it is said. As a result of these pressures, at the rate of approximately 2–6 instances per 100 words (Bortfeld et al. 2001; Fox Tree, 1995; Shriberg, 1994) spontaneous speech contains linguistic elements which are not classically categorized as words or grammatical structures, but instead are variously labeled for example as *interjection of sounds* (Johnson, 1961), *hesitations* (Maclay & Osgood, 1959) and *disturbances* (Mahl, 1956). One type of such element is filled pauses such as *uh* or *um* in English (e.g., *What’s your favorite food? Uh, sushi)*.^1^

Even though early studies saw these types of linguistic elements as markers of hesitation or disfluency, more recent research has identified filled pauses as polyfunctional devices that can show both discourse-structuring and self-repairing uses (Clark & Fox Tree, 2002; Götz, 2013). Thus, filled pause production can be considered to be to some extent voluntary and useful.

A number of corpus-based and experimental studies have investigated how filled pauses are produced and interpreted. However, a central, less explored, debate in the literature concerns how speakers’ minds process and categorize filled pauses, namely, whether filled pauses are non-linguistic, unpredictable and non-systematic vocalizations (e.g., Levelt, 1983; Maclay & Osgood, 1959), fillers with little meaning (Corley & Stewart, 2008), or maybe more like words (e.g., Clark & Fox Tree, 2002; Tottie, 2017) or grammatical elements (Tottie, 2017). This paper takes a usage-based approach and considers whether the frequent co-occurrence of filled pauses with particular words results in the construction of larger linguistic representations. In other words, do those filled pauses that occur regularly with other words combine to form chunks, as happens with other frequently co-occurring words? We will report on three studies. Study 1 reports a corpus analysis on the distributional frequency patterns of filled pauses (*um* and *uh*) in naturalistic data. Based on the results of Study 1, Study 2 investigated acceptability judgments of sentences in which the presence versus absence and the location of filled pauses was manipulated. Lastly, Study 3 was a sentence recall experiment tapping into the underlying representation of filled pauses.

### 1.1 The function of filled pauses in spoken language

While filled pauses have been documented and analyzed since early psycholinguistic research (Goldman-Eisler, 1968; Maclay & Osgood, 1959), their production has only been closely investigated more recently, predominantly using a number of different corpus-based approaches (e.g., Bortfeld et al., 2001; Clark & Fox Tree, 2002; Crible et al., 2017, 2019; Levelt, 1983, 1989; Rendle-Short, 2004; Schneider, 2014; Swerts, 1998; Tottie, 2011, 2015, 2017). Early studies often categorized *um* and *uh* as involuntary noise, which was a result of speakers encountering language production problems rather than having much deliberate function or meaning attached to them, in particular *uh* (e.g., Levelt, 1983, 1989). This was partly based on the observations that filled pauses usually occur in important locations for planning, such as phrase boundaries (Maclay & Osgood, 1959), that utterances that are longer and more complex elicit more hesitations (Goldman-Eisler, 1968; Maclay & Osgood, 1959), and infrequent or complex words are more likely to be preceded by filled pauses (Beattie & Butterworth, 1979; Schnadt & Corley, 2006). However, more recent studies have found that although the length of the utterance predicts the production of other types of disfluency markers, it does not of filled pauses (Fraundorf & Watson, 2008). Thus, even though the retrieval of low-frequency words and the construction of phrases/clauses/sentences is certainly one reason for the production of filled pauses cognitive load issues are not the only reason why filled pauses are produced.

The two filled pause variants (*um* and *uh*) seem to behave differently. The nasal variant (*um*) is associated with strong boundaries, silent pauses and initial position, in other words, with discourse-marking functions, while the non-nasal variant (*uh*) is more typical of utterance-internal position and of weaker boundaries, that is, often used in repair contexts (Fox Tree, 2001; Rendle-Short, 2004; Swerts, 1998). This seems to be the case also in written texts, where *um* occurs less frequently in self-correction contexts than *uh* (Tottie, 2017). Thus, although *uh* is likely to occur with disfluency, *um* could potentially have additional discourse functions.

Studies on adults and children with autism spectrum disorder (ASD) provide further evidence that *um* is not merely a disfluency but can have pragmatic functions. People with ASD produce fewer *ums* relative to their peers but *uh* rates are unaffected (Irvine et al., 2016; McGregor & Hadden, 2020). As pragmatic deficit is a core symptom of ASD, these studies indicate that in particular *um* would have pragmatic functions. That is, *um* can serve a listener-oriented function, whereas *uh* may be more speaker-oriented (i.e., disfluency).

The above studies suggest that filled pauses are relatively frequent in naturalistic language production, but that the two filled pause variants (*um* vs. *uh*) have different functions, ranging from language production functions to discourse functions.

### 1.2 Filled pauses and language comprehension

Mouse-tracking, eye-tracking and event-related potential (ERP) studies have investigated the relationship between filled pauses and reference resolution, in particular by varying the type of words following the filled pause: new versus given information (Arnold et al., 2004; Barr & Seyfeddinipur, 2010); complex versus simple constituents (Watanabe et al., 2008); high- versus low-frequency words (Bosker et al., 2014), high- versus low-predictability words (Corley et al., 2007) and items difficult versus easy to name (e.g., Arnold et al., 2007). All these studies converge on the findings that filled pauses reduce the difficulty associated with new, more complex, low-frequency and low-predictability words. However, some studies (e.g., Corley & Hartsuiker, 2003; Fraundorf & Watson, 2011) have suggested that filled pauses also facilitate comprehension when a filled pause precedes a high predictability word, suggesting that listeners are receptive to the use of filled pauses even with “easy” words. Thus, even though filled pauses may be perceived by listeners as disfluencies in spoken language, they seem to have a function in comprehension whereby the presence of a filled pause helps the listeners process what is being said. Importantly, even though in production the nasal versus non-nasal variant of filled pause seems to have different distributional characteristics, *uh* more typically indicating higher production load than *um*, in comprehension (e.g., Arnold et al., 2004) and in recall (Fraundorf & Watson, 2011), the two variants seem to create similar effects.

The presence of filled pauses has also been found to facilitate the comprehension of repair sequences (e.g., *yel. . .uh. . .orange* vs. *yel. . .orange* for “orange”) (Brennan & Schober, 2001). Brennan and Schober suggest that this might be because the filled pause gives extra processing time for the listener to interpret the repair sequence. However, the facilitating effect of filled pauses on comprehension is not only created by the listener having extra processing time as, for example, coughs that match the location and length of filled pauses hinder recall (Fraundorf & Watson, 2011). Furthermore, silent pauses do not bring about the same effects on judgments of speaker knowledge as filled pauses (Brennan & Williams, 1995). Thus, filled pauses seem to have a different function and representation than silent pauses and non-linguistic vocalizations.

Filled pauses often occur at clause boundaries (e.g., Maclay & Osgood, 1959; Swerts, 1998), and the presence of filled pauses in these types of locations might help listeners disambiguate complex sentence structures that might otherwise be difficult to parse (Bailey & Ferrera, 2003). In longer texts (e.g., when listening to the *Alice in Wonderland* story), the use of filled pauses at clause boundaries where new discourse is introduced has been identified to facilitate plot comprehension (e.g., Fraundorf & Watson, 2008). However, listeners might benefit from the presence of filled pauses even in non-typical locations (e.g., plot medially rather than at plot boundaries) (Fraundorf & Watson, 2011, Experiment 2), thus suggesting that listeners pay attention to filled pauses in a number of locations within sentences and larger units of language.

Even though we know a fair amount about the production and comprehension of filled pauses, to the best of our knowledge, no prior study has tested the effect of filled pauses in native speech when occurring with non-complex high-frequency words onto speakers’ representation and consequent acceptability of filled pauses, a gap that the present study intends to fill.

### 1.3 The representation of filled pauses

The linguistic categorization of filled pauses has been debated in the literature for decades. Based on the early view of filled pauses being just involuntary noise (e.g., Levelt, 1983; Maclay & Osgood, 1959), the assumption was that filled pauses were extralinguistic items. However, the fact that filled pause production can be suppressed for example in public speaking contexts (e.g., Duez, 1982), filled pauses are produced even in written texts (where they are separated from other linguistic items by the use of spaces and punctuation) (e.g., Tottie, 2017), and filled pauses seem to have a different function and representation than silent pauses and coughs (Brennan & Williams, 1995; Fraundorf & Watson, 2011) gives rise to the possibility that some filled pauses might be part of speakers’ language representations. Furthermore, the form and context of filled pause use are subject to language-specific constraints (Crible et al., 2019) indicating that filled pauses are learned from language exposure like other linguistic items.

#### 1.3.1 What types of linguistic elements could filled pauses be?

Clark and Fox Tree (2002) argue that because *um* and *uh* have a meaning, structural constraints on their use and interpretation, and are at least partially planned for, they should be viewed as words, maybe like interjections (see also Norrick, 2015). Biber et al. (1999, p. 1082) take a similar stance and suggest that filled pauses are one type of *inserts* “stand-alone words which are characterized in general by their inability to enter into syntactic relations with other structures.” Tottie (2011, 2015, 2017) investigated the status of filled pauses as words, in particular as pragmatic markers or “planners,” by testing different criteria such as their intentionality, their sociolinguistic variation, their co-occurrence with silent pauses and with other pragmatic markers (*you know*, *I mean* etc.). She argued that even though the basic function of filled pauses is to give speakers time to plan, they have similar uses as other words in the lexicon, and could be viewed as quasi-words (Tottie, 2015) or as adverbs, particularly in writing (Tottie, 2017). She further mentions that, in some contexts, filled pauses can become cliticized with short words such as *and*, *but*, and other function words. This view is shared by Schneider (2014), who considers *and-uh* and *but-uh* as lexicalized chunks serving a time-buying function.

Van Lancker Sidtis and Sidtis (2018) report a positron emission tomography (PET) imaging study investigating the blood flow in the right and left hemisphere during language production, in which they focused on the production of *um/uh*. The study showed that filled pauses were located in the left hemisphere, indicating that *um/uh* are processed/represented similarly to lexical/grammatical items.

To recap, even though a number of studies have investigated filled pauses, the question as to the nature of filled pauses as speakers’ representations still remains. In the current paper, we will adopt the usage-based linguistics viewpoint and investigate the nature of filled pauses.

### 1.4 Usage-based assumptions on language representation and processing

Usage-based linguistics models, such as the schema (e.g., Bybee, 1998, 2006; Bybee & Slobin 1982; Langacker, 2000), constructivist (Goldberg, 2006) and connectionist accounts (e.g., MacDonald & Christiansen, 2002; Plunkett & Marchman, 1993; Rumelhart & McClelland, 1986) assume no qualitative differences between different types of linguistic items (e.g., words vs. grammatical elements) but that linguistic knowledge exists along a continuum from more concrete to more abstract. Furthermore, they see all language representations as form-meaning pairings that are learned in a piecemeal fashion from language exposure, purely by associative mechanisms (e.g., Tomasello, 2003), and are subject to distributional frequency effects throughout the speakers’ life span (e.g., Dąbrowska & Street, 2006).

Input/output is thought to influence how easy/difficult a given linguistic construction is to process and acquire and what the representation is like. Thus, any linguistic item can be processed like a word/grammatical item provided it has a function and is frequent enough for speakers to start building a stable representation of the item.

Type and token frequencies in the input and output are expected to affect the acquisition, processing, and representation of linguistic constructions, but in different ways (Bybee, 1998; MacDonald & Christiansen, 2002). High token frequency (i.e., a construction with little or no variation) is likely to result in a lexically specific representation due to literal similarity (Gentner & Medina, 1998) (e.g., *I love you*, if the speaker experiences the sequence I + love + you frequently) and would be represented as a multiword “chunk” or a “collocation” comprising two or more morphemes (e.g., Bybee, 1998). Experimental and corpus studies suggest that children’s (e.g., Arnon & Clark, 2011; Bannard & Matthews, 2008; Kirjavainen et al., 2009, 2017; Wilson, 2003) and adults’ (e.g., Arnon & Cohen Priva, 2013, 2014; Grimm et al., 2017; Tremblay et al., 2016) language representations contain lexically specific constructions that reflect distributional frequency patterns. Adults are for example faster to recognize (Arnon & Snider, 2010) and produce elicited and spontaneous multiword sequences (Arnon & Cohen Priva, 2013, 2014) that are highly frequent in comparison to less frequent multiword sequences. However, older children and adult speakers do not rely on stored sequences alone but are productive language users, whose abstract linguistic representations come about as a result of high type frequency (i.e., variability within a linguistic construction; e.g., NP-V-NP, if the speaker has experienced a large number of transitive sentence types) (Bybee, 1998) and the process of analogy (e.g., Gentner & Medina, 1998; Tomasello, 2003) when the speaker notices similarities between different exemplars of the syntactic pattern (e.g., *I love you, I love him, I like you, Mary hates Mike*). In between the two lie partially lexically specific constructions, such as productive bound morphemes and clitics (e.g., past tense V-*ed*) in which the frequent occurrence of the morpheme/clitic in a particular slot has resulted in the morpheme/clitic being present in the linguistic representation. Importantly, language representations are organized as a taxonomic network of constructions, and can be seen to exhibit a Russian-doll phenomenon whereby a construction (e.g., *I love you*) can be seen as an instance of a more schematic construction(s) (e.g., NP-V-NP).

In relation to filled pauses, these usage-based assumptions would lead to the following predictions. First, if speakers recognize the co-occurrence of filled pauses with specific words and that there is a discernible function in their use (e.g., politeness, uncertainty), then filled pauses can be expected to have developed a lexically specific representation in that co-occurrence. That is, they will be similar to the chunk I + love + you. Second, if speakers have experienced filled pauses in particular locations but with a number of different words (similarly to the past tense -*ed*) they are likely to have developed a partially lexically specific representation of the filled pause in that location. Third, if speakers have experienced filled pauses with a number of words and in a number of different locations, they should have also built a more abstract representation of filled pauses such that filled pauses can occur with a large number of word types and in a number of locations. If it is the case that there are no clear associations with particular words or locations, this could also indicate that filled pauses are not linguistic items.

### 1.5 The present study

The present study investigates whether the frequent occurrence of a filled pause with particular highly frequent single syllable words, that is, in contexts where the filled pause is likely to function as a pragmatic marker indicating for example politeness or uncertainty rather than a device alleviating a processing issue, has led to the speakers treating these word-filled pause combinations as lexically (partially) specific units.

We first report a corpus analysis that extracted distributional frequency information for the filled pauses *um* and *uh*. We then report a sentence judgment experiment, followed by a sentence recall experiment.

## 2 Study 1: *Um* and *uh* in the Spoken BNC2014

We conducted a corpus analysis that extracted frequent lexical sequences in which filled pauses occur in naturalistic British English discourse. This was to identify potential collocations based on distributional frequency patterns. The collocation lists with frequency information will be used to identify natural (and unnatural) contexts of use for *uh* and *um* and will be the basis of our experimental material selection.

### 2.1 Method

#### 2.1.1 Corpus used

We used the Spoken BNC2014 (Love et al., 2017), the recent spoken component of the British National Corpus recorded between 2012 and 2016. This choice is motivated by the large size of the corpus and its public availability. We restricted the analysis to revised transcriptions of conversations between two native speakers, with no further restrictions in age, gender or social class. This data contain 3,373,258 words.

There are five main transcriptions of filled pauses in the BNC: *er*, *erm*, *eh*, *uh*, and *um* (no hits for *uhm*, *umh*, or *mh*). However, the first two are clearly the most frequent, with 14,606 tokens of *er* (4329.94 per million words, PMW) and 21,946 tokens of *erm* (6505.88 PMW). Overall, this variety of filled pauses appears to be partly due to the transcriber’s preference or to very specific uses (not as filled pauses), and therefore boils down to the binary *uh* versus *um* pair, in concordance with the bulk of the literature where only two variants, a nasal and a non-nasal, are identified. We will use the spellings *uh* instead of *er* and *um* instead of *erm* in order to be consistent with the bulk of the literature on filled pauses.

#### 2.1.2 Searches conducted

By using the online corpus search interface (https://cqpweb.lancs.ac.uk), we extracted frequency information about two-word chunks (bigrams) in the corpus. The output was searched for high-frequency collocates, with a focus on lexical content words, as opposed to interjections or pragmatic markers (e.g., *well*, *so*), which could be used in contexts of hesitation. We also extracted high-frequency words that almost never collocate with filled pauses (⩽ 1 instance found) and should therefore be of relatively low acceptability.

### 2.2 Results

Below we will report on collocation lists both at the right and left of *uh* and *um* in the Spoken BNC2014.

#### 2.2.1 Right collocates of uh and um (filled pause +1)

In the data, 11,144 different word types were found to collocate with the 14,606 tokens of *uh* and 2586 types with the 21,946 tokens of *um*, which shows that there is a lot more variation for the former.

The top 20 most frequent collocates at position Right +1 of *uh* and *um* can be seen in Table 1. It appears that even though the ranking differs, the two filled pauses (*um*, *uh*) have very similar collocates, which mostly consist of pragmatic markers and conjunctions (*well*, *and*, *but*, *so*, *because*, *if*), answer particles (*yeah*, *yes*, *no*), personal pronouns (*I*, *he*, *she*, *you*, *we*, *they*) and WH-pronouns (*what*, *when*, *which*, *where*). Examples 1 and 2 show the most frequent chunk for each filled pause.

**Table 1.** Top 20 right collocates with their collocate and overall frequency.

Rank	uh	um
1	**I** (1,115/142,476)	**and** (1,743/85,171)
2	**well** (283/21,190)	**but** (978/33,398)
3	**yeah** (586 78,029)	**so** (919 33,063)
4	**what** (230/20,823)	**I** (2,064/142,476)
5	**he** (279/28,178)	**yeah** (947/78,029)
6	**and** (588/85,171)	**what** (378/20,823)
7	**no** (233/26,017)	**er** (288 14,606)
8	**but** (267/33,398)	**well** (333/21,190)
9	**so** (257/33,063)	**she** (350/22,913)
10	**you** (555/87,542)	**oh** (415/29,881)
11	**she** (188/22,913)	**he** (357/28,178)
12	**when** (102/10,028)	**which** (92/4,168)
13	**we** (194/24,956)	**we** (294/24,956)
14	**they** (241/34,874)	**because** (125/8,556)
15	**yes** (96/10,446)	**cos** (156/13,056)
16	**sorry** (26/1,344)	**they** (332/34,874)
17	**it** (578/102,795)	**yes** (76/10,446)
18	**maybe** (36/3,055)	**there** (195/19,548)
19	**where** (48/5,029)	**foamy** (2/3)
20	**cos** (96/13,056)	**if** (99/13,929)

Note. The collocates are ranked by log-likelihood, that is, the ratio of the collocate frequency and the overall frequency of the word in the corpus. As a result, rare words such as “foamy” enter the top 20 because of the high proportion of uses where they co-occur with *uh* or *um*. The first number in the brackets is the frequency of the collocation, while the second number is the overall frequency of the word in the corpus).

(1) I need to start peeling more garlic **uh I** need that knife (S38V 1860)
(2) I just think it’s probably beyond repair **um and** you pay so much for a professional to do that (S2UT 461)

The vast majority of these collocates tends to appear in utterance- or clause-initial position as a result of their syntactic nature. This confirms previous findings (e.g., Swerts, 1998) that filled pauses tend to occur at boundaries and mark discourse structure.

Some of these collocates could be related to the concept of disfluency or uncertainty, such as epistemic adverbials or pragmatic markers (especially *well*). In addition, many low-frequency collocates of *uh* and *um* with a high log-likelihood score appear to be multisyllabic, morphologically complex words or words from the formal register. This is particularly the case for *uh*, which collocates with, for example, *outsource*, *uninterrupted*, *awkwardly*, *surreptitiously* or *indigestion*. The occurrence of a filled pause before such words may signal the speaker’s production effort and momentary lack of cognitive resources.

On the other hand, reformulative markers such as *well* are not always used in disfluent contexts, neither are markers of epistemic modality nor morphologically complex words. The presence of grammatical devices (personal, relative and interrogative pronouns) in Table 1 rather suggests the pervasiveness of filled pauses in many different types of contexts.

Finally, in order to find unnatural contexts for filled pause use, we extracted words with a high overall frequency (*N* > 1000) which only collocate once with *uh* or *um*. The types of words are very similar across the two variants and are mainly very basic nouns (*home*, *world*, *week*, *friends*, *day*), adjectives (*long*, *wrong*, *cool*, *bad*, *fine*) or verbs (*bought*, *am*, *says*, *told*). There are two possible reasons for this result. First, the high frequency of these words makes their production quite automatic for the speaker, and this means the extra time provided by a filled pause is not required. Second, these words typically occur in utterance-medial position, which has been shown elsewhere not to be the preferred context of filled pauses.

#### 2.2.2 Left collocates of uh and um (filled pause –1)

Turning to collocates at the left of filled pauses, the figures are quite different, with “only” 8580 different types for *uh* (against about 11,000 at the right) and 10,558 for *um* (much more than at the right). The top 20 most frequent collocates are presented in Table 2. This table does not include word fragments (mid-word interruptions), which were quite high in the list of *uh*.

**Table 2.** Top 20 left collocates with their collocate and overall frequency.

Rank	uh	um
1	**and** (1,078/85,171)	**but** (963/33,398)
2	**but** (463/33,398)	**yeah** (1,342/78,029)
3	**said** (109/9,423)	**and** (1,410/85,171)
4	**‘s** (540/84,900)	**okay** (214/44,702)
5	**yeah** (499/78,029)	**right** (216/8,994)
6	**cos** (124/13,056)	**yes** (217/10,446)
7	**so** (257/33,063)	**so** (453/33,063)
8	**okay** (70/6,873)	**said** (164/9,423)
9	**like** (323/52,501)	**because** (154/8,556)
10	**because** (76/8,556)	**anyway** (72/2,289)
11	**well** (283/21,190)	**then** (194/15,218)
12	**mean** (64/7,087)	**cool** (31/1,092)
13	**yes** (84/10,446)	**about** (144/10,442)
14	**then** (111/15,218)	**like** (485/52,501)
15	**with** (73/13,640)	**with** (118/13,640)
16	**right** (70/8,994)	**had** (114/9,223)
17	**of** (221/40,273)	**fine** (35/1,621)
18	**was** (214/39,595)	**wow** (21/972)
19	**died** (7/375)	**yep** (9/304)
20	**is** (164/29,653)	**excellent** (8/129)

Left collocates seem relatively similar to ones at the right of filled pauses, with once more a large number of pragmatic markers and conjunctions, especially for *uh* (cf. Schneider, 2014). Personal pronouns no longer appear in the list and are replaced by verbs (*said*, *‘s*, *was*, *died*, *is*, *had*),^2^ prepositions (*with*, *of*, *about*) and, for *um* only, adjectives and interjections often used as stand-alone markers of appreciation (*cool*, *fine*, *wow*, *excellent*). The main difference with right collocates is that some words in Table 2 suggest frequent utterance-medial uses of both filled pauses, especially after verbs and prepositions.

Turning to high-frequency words which almost never collocate with filled pauses, we observe similar types of words at the left as at the right and for both variants, especially basic verbs (*try*, *start*, *coming*, *give*, *came*, *must*, *told*).

### 2.3 Discussion

This corpus study showed that the types of words that frequently collocate with filled pauses are fairly regular across positions (left or right) and variant (nasal or non-nasal), with mainly pragmatic markers, personal pronouns and a number of other grammatical words (prepositions, WH-pronouns). Our findings are comparable to previous corpus-based studies insofar as they confirm the association between filled pauses and clause boundaries. However, they also go beyond previous research by showing the specific lexical chunks in which filled pauses occur, looking both at the left and at the right of each of the two variants. Such a fine-grained approach allowed us to identify collocates of filled pauses besides pragmatic markers or planning particles, which have been the focus of previous studies (e.g., Crible et al., 2017; Schneider, 2014; Tottie, 2011).

We will next turn to the experimental investigations of filled pauses, using our corpus analyses as the basis for item selection.

## 3 Study 2: Sentence judgment

Study 2 is a sentence acceptability rating experiment where we investigate the acceptability of *um* (a) with different words and (b) in different locations that speakers frequently versus infrequently encounter *um.* This tests the idea that speakers have lexically (partially) specific representations of filled pauses. It also allows us to determine whether filled pauses contribute to the well-formedness of sentences.

The words that were chosen from the corpus analysis (Study 1) as experimental materials occurred frequently with *uh* and *um*, but the variant used in the experiment was the nasal filled pause (*um*) as the non-nasal variant (*uh*) is more related to disfluency, disruption and hesitation. Thus, *um* is more likely to be perceived as a fully fledged linguistic item (e.g., a word or a grammatical item) than *uh*.

### 3.1 Method

#### 3.1.1 Participants

Twenty-two (14 female) native British English speakers, aged between 18 and 48 years (mean_years_ 28, *SD_years_* = 10) were recruited. They had no known cognitive deficits and had normal or corrected to normal vision. They were given a £5 retail voucher for their participation.

#### 3.1.2 Materials

Sixty test sentences were created based on lexical sequences identified in the corpus analysis (Study 1) as being frequent or infrequent collocations with filled pauses, once pragmatic markers and interjections had been filtered out in order to work with lexical-grammatical elements at the very core of language structure. We selected one target collocation at the left of the filled pause (*said-um*) and one at the right (*um-I*). All other words occurring in the test sentences were relatively frequent (*N* > 100 in the Spoken BNC2014).

##### 3.1.2.1 Said-um

Based on the results of Study 1, we created five sentences testing the acceptability of the collocation *um* following *said*. We compared the acceptability of the strong collocation *said-um* to conditions where the position of the filled pause is not typical (*um-said*), where the filled pause occurs with an infrequent collocate (*thought-um*) and to baseline sentences without a filled pause. The *said* and *thought* sentences were identical apart from (a) the target word (*said* vs. *thought*), (b) the manipulation of the presence/absence of the filled pause, and (c) the location of the filled pause relative to the target word. Table 3 summarizes the five conditions of our design.

**Table 3.** The conditions for *said/thought*.

	Typical position	Non-typical position
Disfluent *said*	*said um* (5)	*um said* (5)
Disfluent *thought*	*thought um* (5)	X
Baseline *said*	*said* (5)	
Baseline *thought*	*thought* (5)	

Examples (3)–(7) illustrate one of the five sentences used in the different conditions. The sentences used were comparable in length (all between 5 and 8 words, 9–3 morphemes long when *um* was omitted from the calculations).

(3) *said um*: Mary said um Edinburgh was beautiful
(4) *um said*: Mary um said Edinburgh was beautiful
(5) *thought um*: Mary thought um Edinburgh was beautiful
(6) *said*: Mary said Edinburgh was beautiful
(7) *thought*: Mary thought Edinburgh was beautiful

In the remainder of this paper, we refer to these sentences as *said/thought* sentences.

##### 3.1.2.2 Um-I

The sentences testing the acceptability of *um* preceding *I* consisted of five sentences different to the sentences used for *said/thought*. Given that *I* is a sentence subject pronoun, to avoid having the filled pause in the sentence-initial position in the collocate condition (*um-I*), and to ensure that the location of *um* in the two test collocates (*said-um* and *um-I*) had similar structural characteristics, we added a sentence-initial adverbial to all sentences in the *I*-set. Thus, in both types of collocate conditions (*said-um* and *um-I*) *um* occurred at a sentence medial boundary (between a main and a subordinate clause or between a temporal adverbial and a sentence, respectively).

The conditions were the same as for *said/thought* with the exception that we included three infrequent sequences in the analysis of the target *um-I.* Two infrequent collocates (*you* and NAME) were used, because the distribution of *I* and *you* is markedly different (*I* usually occurring in subject positions while *you* also occurs in object positions). Thus, we also included proper names (*George, Tom, James, Paul, Ben*) as an additional comparison. Table 4 summarizes the conditions of our design.

**Table 4.** The conditions for *I/you/*NAME.

	Typical position	Non-typical position
Disfluent *I*	*um I* (5)	*I um* (5)
Disfluent *you*	*um you* (5)	X
Disfluent NAME	*um* NAME (5)	X
Baseline *I*	*I* (5)	
Baseline *you*	*you* (5)	
Baseline NAME	NAME (5)	

Examples (8)–(14) illustrate one of the five sentences used in the different conditions. The sentences used were comparable in length (all between 7–10 words, 9–13 morphemes long when *um* was omitted from the calculations).

(8) *Um I*: Last night um I got really angry with Jack
(9) *I um*: Last night I um got really angry with Jack
(10) *Um you*: Last night um you got really angry with Jack
(11) *Um* NAME: Last night um Paul got really angry with Jack
(12) *I*: Last night I got really angry with Jack
(13) *You*: Last night you got really angry with Jack
(14) NAME: Last night Paul got really angry with Jack

##### 3.1.2.3 Filler sentences

In addition to the above test sentences, 85 filler sentences were created. These consisted of three types of sentences:

a) twenty-five sentences with the same words as in the above test sentences, but these filler sentences were ungrammatical (5 × *said*, 5 × *thought*, 5 × *I*, 5 × *you*, 5 × NAME);
b) thirty grammatical sentences that were different in their sentence frames compared to those used as test sentences;
c) thirty ungrammatical sentences with the same words as in fillers (b) above.

As the participants were to make judgments on the acceptability of the sentences they heard, it was important that the sentences sounded as natural as possible. Thus, a female native English-speaking research assistant recorded the test and filler sentences with the Audacity software, creating a sound file for each test sentence (i.e., filled pauses were not spliced into the sentences). The RA rehearsed the sentences, and thus the sentences from a given set were judged to be very similar by the authors. In addition, to ensure that the quality of the filled pauses between sentences/conditions did not differ considerably we conducted an acoustic analysis on *Praat* (Boersma & Weening, 2017) to analyse each filled pause in the test sentences. This analysis checked the quality of the vowel being produced for uniformity in formants (F1 and F2), pitch levels and length. Results of the analysis showed that the vowels produced for the filled pauses were similar in all aspects and are likely to have minimal effect on the judgment of the listener.^3^

##### 3.1.2.4 PowerPoint presentations

Four random orders of the sentences were created, and the audio files entered in those orders on four separate PowerPoint presentations, each audio file in its own slide. In each of the PP presentations, the audio recordings were numbered from 1–145 so that each slide showed a number that corresponded to the linear number of that sentence in the random sequence of sentences in that PP presentation. The participants heard the sentence and saw the sentence number on the slide but did not see the sentence in written form. The PowerPoint slides changed automatically so that the participants had three seconds to give their acceptability rating for each sentence. Participants were randomly allocated to one of the four orders.

##### 3.1.2.5 Answer sheets

An answer sheet was prepared, which had a line representing a 1–9 point Likert scale for each of the 145 sentences. The lines were numbered from 1–145. These corresponded to the sentence numbers, which were depicted linearly, one number per PP slide on the PowerPoint slide. The Likert scale was divided into three color coded groups: score area between 1 and 3 was colored in red, score area between 4 and 6 in yellow and score area between 7 and 9 in green. These color columns had headings: “poor,” “kind of okay,” “good,” respectively.

#### 3.1.3 Procedure

Participants were tested individually in a quiet room at their university or a private home. They were told that they would engage in a sentence judgment task in which they would hear 145 different sentences one at a time and that they would have to rate as how good each sentence sounded on a scale from 1–9 on the answer sheet. The experimenter explained to the participant that there were no right or wrong answers, that the participant would only hear each sentence once and that they would have three seconds to rate the sentence by circling their response on the Likert scale next to the sentence number.

To familiarize the participants with the rating scale, the speed of rating and the procedure overall, before the testing started, two practice sentences were played (*1. There is a cat outside; 2. There a cat outside is*). The test took approximately 17 minutes.

### 3.2 Predictions

In this experiment, we manipulated the presence versus absence of *um*, its position (typical vs. infrequent) and the collocate word (frequent vs. infrequent). Based on the corpus-based findings from Study 1, we expect that the typical position of *um* (*said*-*um* and *um-I*) will be rated higher than the less typical conditions (*um-said* and *I-um*). We also expect that the frequent collocates *said* and *I* will be rated higher than the infrequent *thought* and *you/*NAME. Finally, we can expect an overall negative effect of *um* on ratings such that baseline items (without *um*) will be rated higher than the disfluent items.

### 3.3 Data analysis

**Figure 1.**
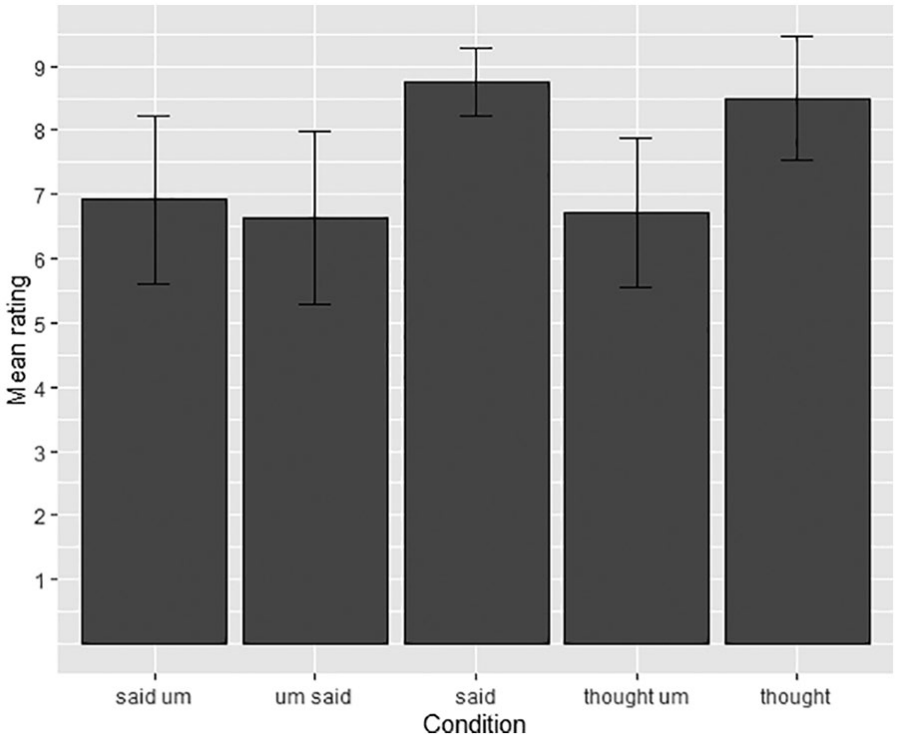
Mean acceptability ratings and standard deviations for the said/thought data.

**Figure 2.**
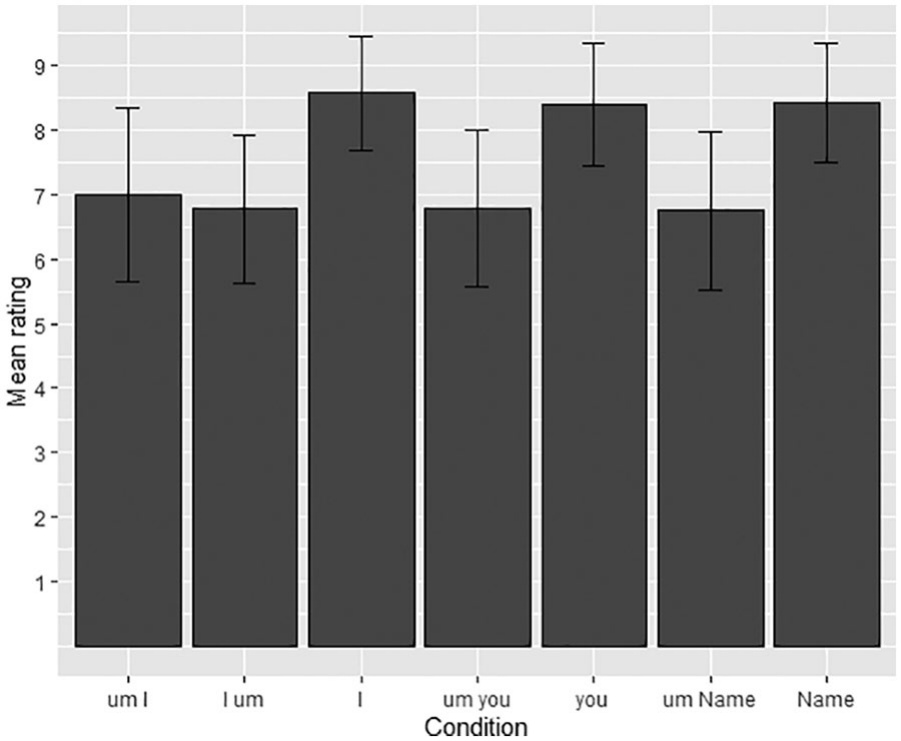
Mean acceptability ratings and standard deviations for the I/you/NAME data.

The regression models were run in R (R version 3.5.2) with the lmerTest package (Kuznetsova et al., 2017). For the ratings analysis, we coded the materials into three categories based on the collocation strength and the related hypothesis: (i) the target collocates *said-um* and *um-I*, which both include high-frequency collocates and the filled pause in its typical position, correspond to the “high” strength category; (ii) the control sequences *um-said* and *I-um* (right word, wrong position) as well as *thought-um*, *um-you* and *um-*NAME (wrong word, right position) correspond to the “low” strength category; (iii) the baseline sequences without any filled pauses correspond to the “none” category. Although we report mean ratings for all five conditions (cf. Figures 1 and 2), the regression analysis will only include collocation strength as an independent variable, with the three levels described previously.

### 3.4 Results

We will report the results for *said/thought* and *I/you/NAME* sentences separately.

Figure 1 presents the mean scores for the five conditions for *said/thought* sequences. Figure 1 shows that the most acceptable sentences were the baseline “collocate-none” sentences that had no filled pause in any location: *said: M* = 8.8, *SD* = 0.53, range: 6–9; *thought: M* = 8.5, *SD* = 0.97, range: 4–9. The collocation (*said-um*) had the third highest acceptability rating (*M* = 6.9, *SD* = 1.3, range: 1–9), followed by wrong-word-right-place (*thought-um: M* = 6.7, *SD* = 1.17, range: 4–9) and right-word-wrong-place (*um-said: M* = 6.7, *SD* = 1.36, range: 2–9).

We performed a linear mixed-effect regression model on the *said/thought* data, with acceptability rating scores as dependent variable. We included the type of collocate as fixed effect (high-frequency collocate, two low-frequency collocates, and two baseline, i.e., collocate-none conditions) and random intercepts per participant and per item (the models with random slopes failed to converge). The final model results are listed in Table 5.

**Table 5.** Regression coefficients on *said/thought* data.

	β	*SE*	*df*	*t*	*p*
Intercept	6.9302	0.1577	36.7866	43.932	< .001
Collocate (low)	–0.2409	0.1104	522.9265	–2.181	< .05
Collocate (none)	1.7091	0.1114	522.9265	15.476	< .001

Table 5 indicates that collocate (low) (i.e., the low-frequency collocates: *um-said* and *thought-um*) were judged significantly lower than the *said-um* sequences, and that collocate(none) (i.e., the baseline with no filled pauses in any location) were judged significantly higher.

Figure 2 illustrates the mean response scores for the conditions testing the target collocation *um-I*. The baseline conditions (*I*, *you*, NAME) had the highest acceptability judgments (*I*-condition: *M =* 8.6, *SD* = 0.88, range: 4–9; *you*-condition: *M* = 8.4, *SD* = 0.95, range: 4–9; NAME-condition: *M* = 8.4, *SD* = 0.92, range: 4–9). The target collocation, *um-I* had the highest mean acceptability rating from the conditions in which filled pauses were present (*M* = 7, *SD* = 1.34, range: 3–9). The low-frequency sequences, *I-um, um-you* and *um*-NAME, were rated slightly less acceptable (*I-um*: *M* = 6.8, *SD =* 1.15, range: 2–8; *um-you*: *M* = 6.8, *SD* = 1.21, range: 2–9; *um*-NAME: *M* = 6.7, *SD* = 1.24, range: 4–9).

A separate model was run for the *I/you/*NAME analysis. The same fixed and random effects were selected as above. The final model results are listed in Table 6.

**Table 6.** Regression coefficients on *I/you/*NAME data.

	β	*SE*	*df*	*t*	*p*
Intercept	7.0052	0.1632	35.9504	42.937	< .001
Collocate (low)	−0.2303	0.1030	746.9424	−2.235	< .05
Collocate (none)	1.4606	0.1030	746.9424	14.176	< .001

Table 6 indicates that, as in the *said/thought* data, the collocate(low) (i.e., the low-frequency collocates: *I-um, um-you, um-*NAME) were judged significantly lower than the *um-I* sequences, and that collocate (none) (i.e., the baseline with no filled pauses in any location) were judged significantly higher.

### 3.5 Discussion

By using a sentence judgment task, Study 2 investigated the acceptability of the collocations (*said-um* and *um-I*) in comparison to low-frequency sequences with *um* and to sequences that contained no filled pauses. We found that sentences in which, according to the corpus findings, the filled pause was in an unusual place (*um-said; I-um*) or collocated with an unusual word were judged as being significantly less acceptable than sentences in which the filled pause might be predicted from the corpus results.

The sentences that did not have filled pauses in any location (baseline) were judged the most acceptable. This is not a surprising result for two reasons. First, given that our corpus analysis found that *said* and *I* occur more frequently without the filled pause than with it (Tables 1 and 2), our experimental data mirrors naturalistic distributional frequency patterns. Second, the highest acceptability of the sentences that did not have a filled pause (in any location) could be also at least partly due to the task used. In an overt judgment task, the ratings are likely to reflect people’s expectation that filled pauses are disfluencies, after all, non-linguists are likely to view filled pauses as undesirable noise (Tottie, 2017, referring to Erard, 2007). Thus, it could be that even though Study 2 found a significant difference between the target collocations and low-frequency sequences, the method did not sufficiently tap into the underlying mental representations of sentences with filled pauses. A further limitation to this study is that we did not include all possible combinations (namely, *um-thought*), which prevented us from testing each factor separately in the statistical analysis. To investigate the representation of filled pauses in a more sensitive task and in contexts where speaker’s expectations as to what the experimenter wants them to say are less overtly present, we conducted Study 3.

## 4 Study 3: Sentence recall

In Study 3, we used a sentence recall task. Sentence recall was thought to be particularly useful for testing the representation of filled pauses as filled pauses can occur in a number of positions within a sentence. Thus, analyzing the recall accuracy rate and the locations in which filled pauses were omitted versus provided should shed light onto speakers’ language representations.

### 4.1 Method

#### 4.1.1 Participants

Twenty-nine (18 female) 18–50-year-old (mean_years_ = 33, *SD*_years_ = 12) native British English speakers with no known cognitive disabilities were recruited, but three of these (all female) were excluded due to them not repeating a single filled pause in any condition - which indicated that they had somehow misunderstood the task (*n* = 2) and due to excessive background noise during the experiment (*n* = 1). None of the participants had taken part in Study 2.

#### 4.1.2 Materials

The study included 95 test sentences, of which 48 were test sentences and 47 were fillers. In addition, 95 distractor math calculations were created.

##### 4.1.2.1 Test sentences

The test sentences were taken from Study 2. However, to allow thorough analyses of the use of *um* in different locations, two more conditions (*um-thought* and *you-um*) with the same sentences as used in the other conditions were added. To manage the length of the test session, the number of sentences per condition was reduced from five to four, and the NAME-condition was deleted from the *I/you*/NAME set.

This results in the factorial design in Tables 7 and 8 for the *said/thought* and *I/you* materials. The new condition for the *said/thought* analysis is illustrated in example (15), and for the *I/you* analysis in example (16).

**Table 7.** The conditions for *said/thought*.

	Typical position	Non-typical position
Disfluent *said*	*said um* (4)	*um said* (4)
Disfluent *thought*	*thought um* (4)	*um thought* (4)
Baseline *said*	*said* (4)	
Baseline *thought*	*thought* (4)	

**Table 8.** The conditions for *I/you*.

	Typical position	Non-typical position
Disfluent *I*	*um I* (4)	*I um* (4)
Disfluent *you*	*um you* (4)	*you um* (4)
Baseline *I*	*I* (4)	
Baseline *you*	*you* (4)

(15) *Um thought*: Mary um thought Edinburgh was beautiful
(16) *You um*: Last night you um got really angry with Jack

##### 4.1.2.2 Filler sentences

Filler sentences consisted of 24 long and/or structurally complex sentences (12 coordinate structures, 12 sentences with relative clauses) and 23 ungrammatical sentences. These were deliberately made long, complex and/or ungrammatical to put strain on the participants’ memory in between test sentences, thus reducing potential priming effects between test sentences.

##### 4.1.2.3 Test and filler sentence manipulation

The new test sentences (*um-thought, you-um*, and new fillers) were recorded by the same native English speaker female as in Study 2. The new recordings as well as those used in Study 2 were manipulated so that each audio file started with a click sound, to indicate to the participant that a sentence was about to be played. There was a 0.5 second pause between the click and the sentence being played and a 2.5 second pause after the sentence.

Four orders were created and participants randomly allocated to one of these. In each of the four orders, the presentation of test sentences was pseudo-randomized to minimize priming effects between the same sentence frame, which was repeated six times (once per condition) for each participant as shown in examples (17)–(22) or between different sentences from the same condition (e.g., *Last night um I got really angry with Jack* vs. *At the party um I had too much to drink*).

(17)* Last night um I got really angry with Jack*
(18)* Last night I um got really angry with Jack*
(19)* Last night I got really angry with Jack*
(20)* Last night um you got really angry with Jack*
(21)* Last night you um got really angry with Jack*
(22)* Last night you got really angry with Jack*

Given that *said/thought* and *I/you* sentences consisted of different sets of sentence frames, they were seen to function as fillers for each other. In addition, the *said*/*thought* and *I/you* sets alternated with an actual filler sentence in between each target (*said/thought* or *I/you*) sentence, as indicated in Example 9.

(9) *Said/thought* > filler > *I/you* > filler > *said/thought* > filler > *I/you*> filler > *said/thought* >

Furthermore, an instance of a given sentence frame (e.g., examples 17–22 above) never occurred as consecutive sentences for a particular set (*said/thought*, *I/you*). That is, there were always a minimum of three different test sentences and four filler sentences in between target sentences from the same sentence frame (e.g., *Last night um I got really angry with Jack* and *Last night I um got really angry with Jack)*.

Each audio file was inserted on a separate slide according to the four pseudo-random orders onto four PowerPoint presentations. No visual information was present in these slides other than the sentence number (e.g., 1).

##### 4.1.2.4 Calculations

To avoid the participant retaining the target sentence in the phonological loop, that is, the vocal component of working memory assigned to rehearsal buffer (Baddeley, 1986), in between hearing a target sentence and repeating it, the participant had to perform a distractor math calculation. All the calculations were additions or subtractions and consisted of triads of slides in the following manner. Two and a half seconds after having heard the target sentence, the participant saw the first math slide which presented a three-digit number (e.g., 183) for three seconds. The slide then automatically changed into a slide that showed only the second part of the calculation consisting of an addition or a subtraction of a number ⩽ 10 (e.g., + 3 =). The fact that the second slide did not contain the initial three-digit number forced the participants to retain the first half of the calculation seen on the previous slide in order to perform the calculation. The participants were instructed to say the answer to the calculation out loud as soon as they knew the answer, but these were not timed. After the participant had given their answer to the calculation, the experimenter pressed a button on the laptop computer keyboard which changed the slide to last slide of the triad that read: *Repeat the sentence* and thus prompted the participant to repeat the sentence they had heard before performing the calculation. Even though the math questions were of similar difficulty (three-digit number ± one-digit number), some of the calculations might have been slightly more difficult than others. Thus, instead of particular calculation always following a particular sentence, the calculations were in the same order in all four PP orders (while the order of the test sentences was different). That is, the calculations were different for different questions in the four orders, thus minimizing any potential effects caused by differences in difficulty between different calculations.

### 4.1.3 Procedure

The participants were individually tested in a quiet room at their university or a private home. They were told that they would hear sentences, some of which would sound more natural than others, but regardless of how unnatural the sentences sounded the participants should repeat the sentences exactly as they heard them. It was emphasized that they should not make any changes to the sentences. They were told that before repeating any given sentence they would have to perform a calculation and give their answer to the calculation. Before the testing started, each participant engaged in one practice sentence–calculation–repetition sequence to familiarize them with the task. The experimenter was present during testing and operated the keyboard as and when needed (after the participant had given their answer to the calculation). The sessions lasted approx. 30 minutes. The sessions were audio recorded. The participants received a £5 retail voucher for their participation.

### 4.1.4 Coding

The experimenter took note of the repetitions during the test situations. Any inaudible or ambiguous sentences were played back after the test session and were transcribed then.

The sentences were coded for being correct or incorrect. Any changes to the target sentence apart from *um* being replaced with the filler *uh* (*n* = 18) were taken as an error.^4^

To investigate the type of errors produced, we further coded the errors into the following categories.

a) Filled pause was moved to a predicted position (error-predicted)
b) Filled pause was moved to an unusual position (error-unusual)
c) Filled pause was omitted (error-omitted)
d) Target verb changed (e.g., *said* was changed for *thought*) (error-wrong verb)
e) Filled pause was produced as in the target sentence, but the participant made lexical changes to the target sentence (apart from changing the target word: *said, thought, I* or *you*) (error-lexical)
f) Several changes in the sentence (e.g., filler moved and a lexical error) (error-several).

To determine reliability for the coding of the responses into the different categories (correct, error-predicted, error-unusual, error-omitted, error-wrong verb, error-lexical, error-several) a research assistant transcribed and second coded 6% of data. The agreement between the two coders was very good (к = .967).

### 4.2 Predictions

In this experiment, we manipulated the same variables as in Study 2, that is, the presence vs. absence of *um*, its position (typical vs. infrequent) and the collocate word (frequent vs. infrequent). We expect that the high-frequency collocates will be repeated more accurately by the participants. In particular, sentences with *um* in a typical position (*said*-*um* and *um-I*) are expected to be recalled better than sentences with *um* in a less typical position (*um-said* and *I-um*). We also expect that the recall of the frequent collocates *said-um* and *um*-*I* will be better than the infrequent *thought-um* and *um*-*you*. Finally, we can expect an overall negative effect of *um* on accuracy such that baseline items (without *um*) will be more accurately recalled than the disfluent items.

### 4.3 Data analysis

All tests were run in R (R version 3.5.2) with the lmerTest package (Kuznetsova et al., 2017). For the accuracy analysis, we used forward model selection with the *anova* function (Baayen et al., 2008), based on our factorial design, as opposed to the effect coding in three categories previously used in Study 2.

### 4.4 Results

**Figure 3.**
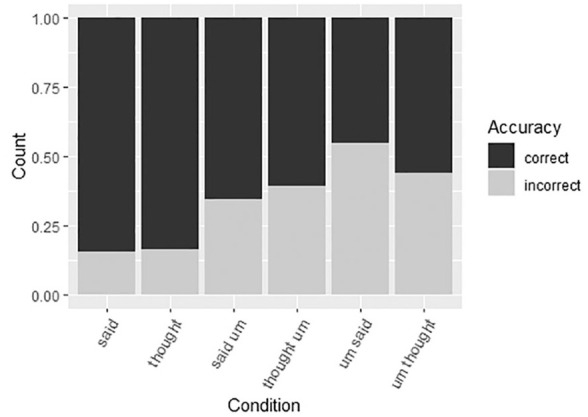
Accuracy rates for said/thought sentences.

**Figure 4.**
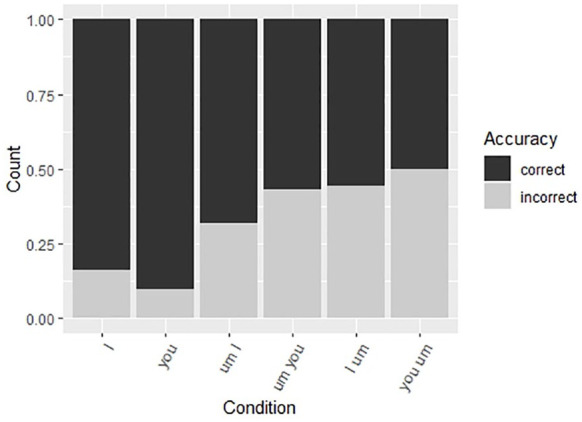
Accuracy rates for I/you sentences.

We first carried out an analysis of accuracy. This was done separately for *said/thought* and *I/you.* Figures 3 and 4 show the rates of accurate versus inaccurate repetitions per conditions for both groups of materials. We see an increasing rate of inaccurate repetitions from baseline sentences (around 80% accurate) to typical positions of the filled pause (*said um, thought um, um I, um you*, around 60% accurate) and non-typical positions, with up to 50% of inaccurate repetitions for *you um* (wrong word, wrong position) and 55% for *um said* (right word, wrong position).

We then ran logistic mixed-effect regression models. On the *said/thought* data, the model with only random effects by participant and by item was significantly improved by adding Presence of filler (Δχ² = 57.263, Δ*df* = 1, *p* < .001) and Position of filler (Δχ² = 7.8862, Δ*df* = 1, *p* < .01) as predictors. Adding the frequency of the collocate did not further improve it (Δχ² = 3.4402, Δ*df* = 3, *p* = .33). The final model returns a main effect of Presence of filler and of Position (see coefficients in Table 9 for *said/thought)*. We see that the presence of a filled pause overall increases the likelihood of an inaccurate repetition in comparison to sentences without a filled pause, but the typical position of the filler (i.e., after *said*, as in the target collocation *said-um*) decreases the likelihood of inaccurate repetitions.

**Table 9.** Regression model for *said/thought* sentences.

	β	*SE*	*z*	*p*
Intercept	−1.9430	0.2982	−6.515	< .001
Filler (present)	1.9195	0.2579	7.442	< .001
Position (typical)	−0.6096	0.2178	−2.799	< .01

We next analyzed the accuracy for *I/you* sentences. The model with only random effects by participant and by item was significantly improved by adding Presence of filler (Δχ² = 65.624, Δ*df* = 1, *p* < .001) and Position of filler (Δχ² = 4.4637, Δ*df* = 1, *p* < .05) as predictors. Adding the frequency of the collocate was only marginally better (Δχ² = 6.3921, Δ*df* = 3, *p* = .09), so we left this predictor out. The final model returns a main effect of Presence of filler and of Position (see coefficients in Table 10 for *I/you*), such that the presence of a filled pause increases the likelihood of an inaccurate repetition, while the typical position of the filler (i.e., before *I*, as in the target collocation *um-I*) decreases the likelihood of inaccurate repetitions.

**Table 10.** Regression model for *I/you* sentences.

	β	*SE*	*z*	*p*
Intercept	−2.1071	0.2992	−7.043	< .001
Filler (present)	1.9805	0.2646	7.486	< .001
Position (typical)	−0.4475	0.2121	−2.110	< .05

**Figure 5.**
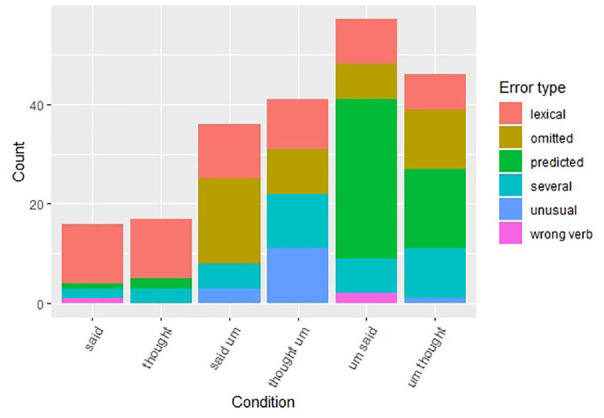
Error responses for said/thought sentences.

Next, we investigated the error patterns. Figure 5 shows that baseline sentences with *said/thought* without a filled pause show similar error patterns, which consists predominantly of lexical errors. However, collocation *said-um* and the wrong-word-right-place variant (*thought-um*) showed different error patterns, as did right-word-wrong-place (*um-said*) and wrong-word-wrong-place (*um-thought*) variants. The most common error in the *said-um* sequence was the omission of the filled pause, which was significantly more common than in *thought-um* sentences (*z* = 2.34, *p* < .05). In *thought-um* sentences the most common error was to move the filled pause to an unusual position or to make several changes to the sentence. When *um* occurred with the right word, but in the wrong place (*um-said*) it was overwhelmingly most commonly moved to the predicted position (to follow *said*). Moving *um* from the pre-verbal to the post-verbal position was not as common with the *um-thought* variant (*z* = 2.16, *p* < .05). That is, the post-*thought* position did not pull as strongly as the post-*said* position. The wrong-word-right-place (*thought-um*) data shows that the error types were balanced over four types of errors, suggesting that participants noticed there was something unusual in the sentence but were not exactly sure what it was.

**Figure 6.**
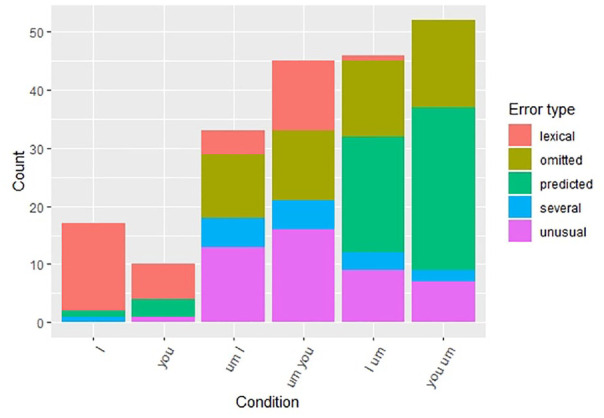
Error responses for I/you sentences.

When it comes to *I/you* sequences, Figure 6 shows that, the pattern is not as clear as for *said/thought* sequences. First, the baseline sentences (*I* and *you* sentences without *um*) show similar error patterns, which consist predominantly of lexical errors. Both the collocation *um-I* and wrong-word-right-place sequence (*um-you*) show a pattern whereby there are a lot of omissions and production of *um* in unusual positions. The omissions could suggest that these sequences are entrenched (i.e., the participants did not notice the filler), but the unusual position errors suggest the contrary, namely, the participants noticed the filler but were not sure where to put it. The right-word-wrong-place sequence (*I-um*) and wrong-word-wrong-place (*you-um*) showed similar error patterns in that the participants moved the filled pause into the predicted pre-pronoun position. These results suggest that there are no lexical effects, but *um* is more natural before the pronoun than after, regardless of the pronoun.

### 4.5 Discussion

To investigate the representation of *um* in the minds of native English speakers, we conducted a sentence recall experiment and analyzed adult speakers’ repetition accuracy, and the error types produced in sentences with and without filled pauses. Our method taps into processing of items that have at least on some level been stored, thus giving an indication of the speakers’ language representations. We found that wrong-place sequences (*um-said, um-thought*) reduced the repetition accuracy and that when *um* was heard in an *um-said* sequence, it was commonly moved to the more usual (*said-um*) position, while no such effect was observed for the *um-thought* sequence. This suggests that *um* was more natural in the post-*said* position than in the post-*thought* position. Moreover, the fact that *um* was more often omitted from *said-um* than *thought-um* sequences indicates that the *um* was so natural in the former that participants did not even notice it there. These results closely mirror the corpus analyses (Study 1) and suggest that *um* is associated with a location where it follows a particular verb.

Contrary to our corpus analysis identifying *um-I* as a collocation, the recall results were not as clear for the *I/you* set and suggested that the representation of *um-I* sequence was no stronger than that of the *um-you* sequence. One explanation for this could be the acoustic resemblance of the filled pause *um* with the first-person verb form *am*. This, in combination with the test materials containing ungrammatical sentences as fillers might have resulted in participants assuming that when they heard *um* they were actually hearing *am* (e.g., *Last night I am got really angry with Jack* for *Last night I um got really angry with Jack).* This could explain why a relatively high number (13) of the errors were produced in the collocate *um-I* condition in which the participants moved the filled pause from the predicted pre-*I* position to the unusual post-*I* position. Alternatively, it could be that *um-I* sequences are associated with sentence-initial positions only, for example in response to questions (e.g., Q: *Are you coming to the party tomorrow?* A: *Um I haven’t decided yet, I might*). The fact that we added sentence-initial adverbials (e.g., *last night, at the party, on Monday*) to the *I/you* sentences might have resulted in *um-I* sequences occurring in unusual locations, contributing to the less clear results found. Lastly, the discrepancy in the results between the pre- (*um-I*) and post-collocate position (*said-um*) could be taken to suggest that *um* in a post-word position creates a stronger representation, possibly because in pre-word positions filled pauses commonly function as a disfluency marker or because post-word position creates a stronger structural representation due to its place being the same as that of clitics and grammatically bound morphemes. In any case, our results suggest that distributional frequency patterns can result in filled pauses forming chunks with particular words, but that distributional frequency might not result in equally strong effects for all co-occurrences.

It is worth pointing out that the baseline sentences (without filled pauses) had significantly higher repetition accuracy than the sentences in which there was a filled pause, and that other than a small number of sentences (*said/thought*: 3, *I/you*: 4), the participants did not add a filled pause to the predicted position in the baseline sentences. In addition to frequency-based reasons, the difficulty associated with sentences that contained collocations relative to the baseline sentences could be explained by the assumption that the presence versus absence of a filled pause in an experimental sentence is likely to be relatively salient to the participant. This in combination with the fact that filled pauses can occur in a number of locations within a sentence (indicating e.g., disfluency or pragmatic meanings) is likely to have created competition between the different possible locations (or if entrenched, the omission of the filled pause) in the sentences that contained filled pauses, while there was no such competition for the sentences in which no filled pause was present.

## 5 General discussion

To shed light on the representation of filled pauses as linguistic (e.g., Clark & Fox Tree, 2002; Tottie, 2017) versus non-linguistic (e.g., Levelt, 1983; Maclay & Osgood, 1959) items, we investigated distributional frequency patterns of filled pauses in a spoken corpus (Study 1) and conducted sentence acceptability judgment (Study 2) and sentence recall (Study 3) experiments on the nasal variant of the filled pause (*um*) in adult native speakers of English. As far as we are aware, the current study is the first investigating filled pauses from this perspective. Our acceptability judgment study found that high-frequency *um* sequences (*said-um*, *um-I*) were rated significantly more acceptable than low-frequency *um* sequences (either right word in the wrong place or wrong word in the right place). Our recall study found that the repetition accuracy of both *said/thought* and *I/you* sentences was affected by the location of *um*, the repetition accuracy being higher for those sentences in which *um* was in a typical (*said/though-um; um-I/you*) versus non-typical position. Furthermore, when *um* was heard in the low-frequency position with the verb *said* (*um-said*), participants most commonly moved it to the high-frequency position (*said-um*), while no such effect was observed for the *um-thought* (wrong-word-wrong-place) sequence. That is, there was more pull for *um* to be produced in the slot following *said* than in the slot following *thought*. This suggests that the verb *said* followed by the filled pause forms a chunk (*said-um*). Our recall study also suggests that wrong-position (e.g., *um-said*) sequences are less acceptable than wrong-word sequences (*thought-um*) indicating that in addition to the lexically specific representation (*said-um*) *um* also has a more abstract syntactic representation, in which it occurs in clause boundaries between main verbs (denoting mental states or communication) and sentential complements. This is in line with previous studies that have found that *um* is associated with strong boundaries (Maclay & Osgood, 1959; Swerts, 1998; Rendle-Short, 2004).

The current paper complements the large body of corpus analyses reporting that filled pauses have a predictable distribution (e.g., Crible et al., 2017; Fox Tree, 2001; Rendle-Short 2004; Swerts, 1998; Tottie, 2017) and links the distribution of filled pauses in naturalistic spoken interaction to speakers’ behavior in experimental contexts. Given that our results suggest that filled pauses can show frequency effects, and form lexically specific chunks and more abstract schemas, our results are consistent with the view that filled pauses, at least in some locations and with some words, can be seen as linguistic items (e.g., Clark & Fox Tree, 2002; Tottie, 2017). Despite our experiments being relatively low-powered (i.e., a few items per condition, 22–29 participants per study), which might prevent the generalization of our results beyond the sample analyzed here, we believe our findings have important theoretical implications.

### *5.1 Said-um* versus *um-I*

Even though the overall results for both collocations (*said-um* and *um-I*) point in the same direction, the results for the *said-um* sentences were stronger than for the *um-I* sentences. This could be explained by the fact that the proportional frequency of *um-I* relative to instances of *I* not being preceded by *um* (or *uh*) in the corpus was smaller (1.45%, 2064/14,2476) than the frequency of *said-um* relative to *said* not being followed by *um/uh* (1.74%, 164/9423). Moreover, while the wrong-word-right-place control sequence (*thought-um*) for the *said-um* collocation was not among the 20 most frequent left collocates, the control sequence for *um-I* (i.e., *um/uh-you*) was among the 20 most common right collocates (0.63%, 555/87,532). This difference in distributional frequency patterns between *said-um* versus *said* when compared to the difference between *um-I* versus *I* and absence of a relatively similarly behaving control word (*thought* vs. *you*) might, to some extent, explain why the *I/you* set showed weaker effects than the *said/thought* set. The distributional frequency differences between the *said/thought* and *I/you* sets and the weak two-word association found for *um-I* could indicate that the representation of *um* with *I/you* is more abstract (e.g., *um*-subject rather than *um-I*) than with *said* (*said-um*). Having said that, the co-occurrence of *um* with specific words might not be the only explanation for the difference found. First, given that previous research has identified strong boundaries as typical filled pause locations (e.g., Rendle-Short, 2004; Swerts, 1998), we created our test sentences so that in the right-place condition (*said/thought-um; um-I/you*/NAME) for both *said/thought* and *I/you*(/NAME) sets *um* occurred at a major boundary. However, due to the characteristics of the collocations, the filled pause in the *um-I/you(/*NAME) sentences occurred in between an adverbial (e.g., *tomorrow, on Monday*) and a pronoun/name while in *said/thought-um* sequences *um* occurred in a boundary between a main clause verb and a sentential complement, which might be a more typical or a stronger boundary thus yielding clearer results. Second, to investigate if the location of the filled pause had an effect on the representational strength, our materials included one right collocate (*um-I*) and one left-collocate (*said-um*). Thus, the location of the filled pause relative to the co-occurring word was different in the two sequences. This might have affected the representational strength (as we explain below).

### 5.2 Are filled pauses more like lexical or grammatical items?

Suggestions have been put forward that filled pauses could be considered lexical, “quasi-words,” “planners” or, in the written language at least, stance adverbials (e.g., Fox Tree & Schrock, 2002; Tottie, 2011, 2015, 2017) or grammatical elements (when cliticized, as in *anduh* or *butuh*). The current study can contribute to this debate. First, if we assume that filled pauses are adverbials (e.g., Tottie, 2017) or interjections (Clark & Fox Tree, 2002) then one would assume that the location of filled pauses would be relatively free (even in the context of strict word order languages like English). But this is not what we found. In the sentence recall study (Study 3), the sentences in which the filled pause was in a typical position had higher repetition accuracy. This indicates that it is not just the presence of the filled pause, but it is its exact location that is important. This could be interpreted that filled pauses are similar to grammatical items such as suffixes, clitics or prepositions in that their location within a sentence is relatively rigid.

Second, in the sentence recall task the participants often omitted filled pauses in *said-um* (collocate) sentences suggesting that the participants did not notice or recall that the filled pause was there. In this respect, filled pauses are similar to grammatical items that speakers often ignore and omit when repeating materials (speakers retain the semantic information rather than the surface form).

Third, as mentioned previously, filled pauses and the words they frequently occur with form the strongest lexically (partially) specific chunks when the filled pause occurs after the collocate (*said-um*) rather than before it (*um-I*) even when in both cases the filled pause occurred at a major boundary. This could be related to the fact that disfluencies often occur before words (e.g., Arnold et al., 2004; 2007; Barr & Seyfeddinipur, 2010; Watanabe et al., 2008) and consequently, if a filled pause frequently occurs in a location that is not associated with disfluency (i.e., after a particular word) it might create a clearer function-based association between the filled pause and the word it follows. Furthermore, grammatical morphemes and clitics in English occur in word final positions, and similarly to filled pauses are often semantically relatively light (e.g., 3psg *-s*; the past tense -*ed* when produced with a temporal adverbial) and if speakers frequently experience *um* in the suffix-position, they might, via analogy, start processing the filled pause similarly to a grammatical element. This suggests that it is not just the location of *um* relative to sentence boundaries but also the location of it relative to words it occurs with. However, given that we only included one collocate for a given location (*said-um* vs. *um-I*) future research needs to establish if a larger sample of left versus right collocates of *um* replicate this effect.

Based on the above points, our results provide evidence for Tottie’s (2017, p. 125) suggestion that, in the spoken language, filled pauses, when they follow other monosyllabic words like *and, but, when, had*, etc., can be seen as being similar to grammatical items, like suffixes or clitics.

## 6 Conclusion

The current study tested if, in addition to being related to disfluency, filled pauses (*um*) can function like fully fledged linguistic items. We found that when a filled pause followed its collocate, speakers had lexically specific chunks and partially specific chunks with *um*. This in turn was taken as evidence that filled pauses can function as linguistic items, similar to grammatical bound morphemes or clitics.

## Notes

1. In order to be consistent with the bulk of the literature on filled pauses, we will be using the spellings *uh* instead of *er* for the non-nasal variant and *um* instead of *erm* for the nasal variant of filled pauses.

2. Note that *’s* could also be a possessive suffix.

3. All materials, the full list of filled pause qualities, data and R-script for Study 2 and Study 3 are available at https://osf.io/fkxa4/.

4. We ran the model with the data where verbatim sentences with *uh* instead of *um* were coded as an error, and the results were more or less identical to the results where replacing *um* with *uh* was not seen as an error.
